# The benefits of regular aerobic exercise training on cerebrovascular function and cognition in older adults

**DOI:** 10.1007/s00421-023-05154-y

**Published:** 2023-02-19

**Authors:** Edward S. Bliss, Samia M. Biki, Rachel H. X. Wong, Peter R. C. Howe, Dean E. Mills

**Affiliations:** 1 Respiratory and Exercise Physiology Research Group, School of Health and Medical Sciences, University of Southern Queensland, Toowoomba Campus, 11 Salisbury Rd, Ipswich, QLD 4305 Australia; 2 Centre for Health Research, Institute for Resilient Regions, University of Southern Queensland, Ipswich, QLD Australia; 3 School of Biomedical Sciences and Pharmacy, Clinical Nutrition Research Centre, University of Newcastle, Callaghan, NSW Australia; 4 Adelaide Medical School, University of Adelaide, Adelaide, South Australia Australia

**Keywords:** Cardiometabolic, Aerobic exercise training, Cerebrovascular function, Cognition, Ageing

## Abstract

We compared the differences in cerebrovascular and cognitive function between 13 aerobic exercise trained, older adults and 13 age-, height- and sex-matched sedentary, untrained controls. We determined whether other measures accounted for differences in cerebrovascular and cognitive function between these groups and examined the associations between these functions. Participants undertook anthropometric, mood, cardiovascular, exercise performance, strength, cerebrovascular, and cognitive measurements, and a blood collection. Transcranial Doppler ultrasonography determined cerebrovascular responsiveness (CVR) to hypercapnia and cognitive stimuli. The trained group had a higher CVR to hypercapnia (80.3 ± 7.2 vs 35.1 ± 6.7%, *P* < 0.001), CVR to cognitive stimuli (30.1 ± 2.9 vs 17.8 ± 1.4%, *P* = 0.001) and total composite cognitive score (117 ± 2 vs 98 ± 4, *P* < 0.001) than the controls. These parameters no longer remained statistically different between the groups following adjustments for covariates. There were positive correlations between the total composite cognitive score and CVR to hypercapnia (*r* = 0.474, *P* = 0.014) and CVR to cognitive stimuli (*r* = 0.685, *P* < 0.001). We observed a relationship between cerebrovascular and cognitive function in older adults and an interaction between regular lifelong aerobic exercise training and cardiometabolic factors that may directly influence these functions.

**Supplementary Information:**

The online version contains supplementary material available at 10.1007/s00421-023-05154-y.

## Introduction

Normal ageing is accompanied by a decline in fluid cognitive ability (ability to respond to novel situations compared with acquired or crystallised knowledge), which is preceded by reduced cerebrovascular structure and function (Harada et al. 2013; Kennedy and Raz 2015; Bangen et al. 2014; Barnes et al. 2012; Rogers et al. 1985; Ainslie et al. 2008; Salthouse 2012; Bliss et al. 2021). This reduction may be attributed to modifiable risks factors that determine cardiometabolic health, including nutrition and physical activity (Toda 2012; Woods et al. 2011; Bangen et al. 2014; Toth et al. 2017; AIHW 2012; Bliss et al. 2021). As cerebrovascular function and cognition decline during ageing, our ability to perform general tasks associated with daily life decreases (Paterson et al. 2007; Taylor 2014; Bliss et al. 2021; Salthouse 2012). Given that the population is rapidly ageing, and the costs associated with loss of independence and chronic diseases on healthcare systems are rapidly increasing (Taylor 2014; Brown et al. 2017), it is imperative that evidence-based strategies to prevent and reduce the decline in cerebrovascular function and cognition are identified, such as aerobic exercise.

Regular exercise has also been shown to help maintain cerebral perfusion in healthy ageing (Ainslie et al. 2008; Rogers et al. 1990; Bailey et al. 2013), which would be an important, simple and cost-effective treatment. For example, Ainslie et al. (2008) reported that the normal age-related decline in mean cerebral blood flow (CBF) velocity (CBF_V_) in the middle cerebral artery (MCA) was 0.38–0.45% per year between the ages of 18 and 80 years. However, individuals who regularly engaged in at least 150 min of moderate-vigorous intensity aerobic exercise training for at least 2 years (self-reported data and objectively determined using maximal aerobic exercise capacity measurements) demonstrated a 17% higher CBF_V_ across the lifespan compared to age-matched sedentary counterparts (Ainslie et al. 2008). Nevertheless, a limitation to this study was that it did not measure the effects of regular physical activity and exercise on CVR. CVR is the ability to modify regional blood flow in response to specific physiological (e.g. hypercapnia) or psychological stimuli (Willie et al. 2011; Serrador et al. 2000). It is the rapid increase in CBF_V_ in a conduit cerebral blood vessel which is attributable to dilatation of the microvasculature downstream in response to local chemical changes induced by physiological or psychological stimuli (Willie et al. 2011). CVR is an important measure as the cerebral vasculature is highly responsive to its environment, particularly to the partial pressure of arterial carbon dioxide, and to changes in neuronal metabolism. Further, it is the responsiveness of the cerebrovasculature that maintains both cerebral autoregulation and neurovascular coupling (NVC), and, consequently, cerebral function (Miller et al. 2018; Braz et al. 2017; Toth et al. 2017; Duchemin et al. 2012).

Cross-sectional research that has measured the difference in CVR between aerobically exercise trained and untrained individuals has shown mixed findings. Barnes et al. (2013 Barnes et al. (2013) and Marley et al. (2020) reported that CVR to hypercapnia was positively associated with maximal aerobic exercise fitness and Bailey et al. (2013) reported a positive linear relationship between maximal aerobic exercise fitness (measured objectively using maximal exercise capacity tests), CBF_V_ and CVR. However, others have reported no difference in CVR between aerobically exercise trained and untrained individuals (Braz et al. 2017; Miller et al. 2018) or a lower CVR in elderly Masters athletes compared to untrained controls (Zhu et al. 2013; Thomas et al. 2013). The difference in findings is not clear, but may be due to differences in the technique used for testing CBF (Braz et al. 2017; Joris et al. 2018; Willie et al. 2011), variation in ages and training status (Miller et al. 2018), use of subjective or objective measurements to determine exercise training status and capacity, choice of which cranial or extracranial vessel is used for testing (Braz et al. 2017; Willie et al. 2011) or difference in hypercapnia protocols and the derivatives of CVR (Miller et al. 2018).

Maintaining optimal cerebrovascular function may also delay cognitive decline (Bangen et al. 2014; Toth et al. 2017). Cognitive decline throughout the lifespan is well defined and previous research has reported that middle-aged to older adults who participate in regular physical activity and aerobic exercise training or have undertaken an aerobic exercise training intervention (either self-reported or objectively measured using exercise capacity tests) have greater cognitive capacity than sedentary individuals (Anderson-Hanley et al. 2012; Baker et al. 2010; Bossers et al. 2015; Hoffmann et al. 2016; Lautenschlager et al. 2008; Sobol et al. 2016; Vreugdenhil et al. 2012). However, these studies do not typically measure overall cognitive capacity and focus predominantly on one or few cognitive domains, such as executive function or working memory. The focus on more short-term exercise studies is probably associated with the poor compliance and adherence observed with chronic exercise training (Ngandu et al. 2022; Lam et al. 2015). Therefore, this also reduces the ability to determine the long-term impact of chronic exercise interventions on cognition and cerebrovascular function through both intervention trials and longitudinal studies. This highlights a current knowledge gap between short-term changes and chronic adaptations and whether the ‘gap’ can be closed by implementing exercise training regimes. None of these studies have examined the association between cognition and cerebrovascular function.

Only two cross-sectional studies have measured and associated cognition with cerebrovascular function (Rogers et al. 1990; Brown et al. 2010). These studies demonstrated that older adults with higher aerobic fitness had greater cerebrovascular and cognitive function compared to sedentary individuals. However, CVR to psychological stimuli (NVC) was not tested, nor were any correlation analyses between cerebrovascular and cognitive function performed. Here it would be expected that higher cognitive function would be associated with increased cerebrovascular function, while lower cognitive function would be associated with reduced cerebrovascular function. This is important as it measures complex interactions between neuronal metabolic demands and local haemodynamic changes, thus ensuring that the metabolic demands of the brain are met immediately by the vasculature, within the first few seconds of these metabolic demands increasing (Duchemin et al. 2012; Toth et al. 2017). Further, other potential covariates including body composition, cardiovascular function, biochemistry and educational level that may also explain the differences in cerebrovascular and cognitive function between aerobic exercise trained and untrained sedentary individuals were not accounted for. These important variables may directly impact vascular, cerebrovascular and cognitive functions and may also explain the differences between aerobic exercise trained and sedentary, untrained individuals (Bliss et al. 2021). This is because ageing is associated with reductions in vascular, cerebrovascular and cognitive functions and these potential covariates along with sedentary behaviour exacerbate their decline by essentially promoting low-grade systemic inflammation, oxidative stress and reducing endothelial function (see Bliss et al. (2021) for in depth review of mechanisms) (Bangen et al. 2014; Toth et al. 2017; Toda 2012; Woods et al. 2011).

Accordingly, the aims of our study were threefold. First, to compare the differences in cerebrovascular and cognitive function between older adults that had undertaken regular aerobic exercise training and those that were sedentary and untrained. Second, to determine the association between cerebrovascular and cognitive function in these individuals. Finally, to examine if other measures including body composition, cardiovascular function, biochemistry and educational level would account for the differences between trained and untrained groups. We hypothesised, first, that CVR and cognition would be higher in the trained group compared with the untrained group. Second, cerebrovascular and cognitive function would be correlated, thus reflecting an interdependent relationship between these functions.

## Methods

### Participants

Thirteen aerobic exercise trained older adults and thirteen age-, height- and sex-matched sedentary, untrained controls participated in the study (Table 1). These participants were recruited from South East Queensland, Australia, which includes metropolitan, regional and rural areas, between October 2019 and March 2021. Trained participants were defined as those who had regular participation in at least 150 min of moderate-vigorous intensity aerobic exercise training per week consistently for at least 2 years and untrained participants as those who were physically inactive for at least the previous 2 years (AIHW 2018). The trained individuals were recruited from various sporting clubs, such as road-runner associations, cycling clubs, swimming clubs, gymnasiums, bushwalker clubs and the general public who participate in aerobic exercise but are not associated with any particular sport or club. This was to ensure that we had a mix of participants who undertook different types of aerobic exercise over the lifespan, as the purpose of the study was not to focus on a single mode or type of aerobic exercise. It should be noted that recruitment and testing were terminated earlier than anticipated due to the impacts of COVID-19 and the restrictions placed on gathering, research and travel by the Queensland and Australian Governments. The exclusion criteria were as follows: aged under 50 years or over 80 years; diagnosed cognitive impairment, current smokers; blood pressure ≥ 160/100 mmHg; prescribed insulin, hormone-replacement therapy, or oral anticoagulants; and a significant history of cardiovascular, neurological, cerebrovascular, kidney, liver disease or cancer. Participants were only included in the study if they were on a stable medication treatment plan for conditions that did not contradict the exclusion criteria such as hypertension, dyslipidaemia, type two diabetes and osteoarthritis. Statin therapy was also included in this study, despite the controversy that prolonged statin therapy may reduce both muscle and cognitive function, as no clear conclusions regarding this have been determined (see (Ruscica et al. 2022) for detailed comparison). The Yale Physical Activity Survey (YPAS) (Dipietro et al. 1993), Lifetime Physical Activity Questionnaire (LPAQ) (Friedenreich et al. 1998), and a customised health and wellbeing screen were initially used to determine whether participants met the study criteria, as well as their exercise training status. The LPAQ assessed the types of and length of participation in various physical activities throughout the lifespan and is reported in Table 1 as consistent years exercise training. The YPAS assessed current physical activity status semi-quantitatively including energy expenditure and duration of exercise and is also reported in Table 1. A higher score in these surveys is considered favourable, with the exclusion of sedentary behaviours, such as the sitting index score. All study procedures were approved by the University of Southern Queensland Research Ethics Committee (H19REA015), which adheres to the Declaration of Helsinki*.* The study was registered with the Australian and New Zealand Clinical Trial Registry (ACTRN12619001291178). All participants provided written, informed consent prior to participation in the study.

**Table 1:** Participant demographics, anthropometrics, body composition, grip strength, exercise performance, nutritional intake, exercise performance and mood for the untrained and trained groups

Variable	Untrained (*n* = 13)	Trained (*n* = 13)	*P* value
Demographics			
Age (years)	66 ± 2	64 ± 2	0.471
Sex (Male/Female)	6/7	6/7	–
Education (years)	16 ± 1	21 ± 1	**0.002**
Retired (Yes/No)	8/5	8/5	–
Consistent exercise training (years)	0	40 ± 4	** < 0.001**
Anthropometrics			
Body mass (kg)	99.3 ± 4.9	70.1 ± 4.7	** < 0.001**
Height (m)	1.69 ± 0.02	1.70 ± 0.03	0.830
Body mass index (kg/m^2^)	34.6 ± 1.5	24.0 ± 1.2	** < 0.001**
Hip circumference (cm)	124 ± 4	101 ± 3	** < 0.001**
Waist circumference (cm)	115 ± 3	86 ± 3	** < 0.001**
Hip-to-waist ratio	0.93 ± 0.02	0.86 ± 0.01	**0.003**
Body composition			
Total lean mass (kg)	52.8 ± 3.0	47.2 ± 3.0	0.212
Total body fat (%)	45 ± 2	28 ± 3	** < 0.001**
Total bone mineral density (g/cm^2^**)**	1.31 ± 0.03	1.26 ± 0.05	0.335
Total bone mineral content (g/cm)	2876 ± 158	2690 ± 186	0.457
Grip strength			
Dominant hand (kg)	32.0 ± 3.0	32.3 ± 2.8	0.801
Non-dominant hand (kg)	30.0 ± 2.5	30.2 ± 2.5	0.949
Exercise performance			
6-min walk test distance (m)	487 ± 19	630 ± 21	** < 0.001**
Nutritional intake			
Total energy intake (kcal)	2538 ± 250	2735 ± 272	0.600
Yale physical activity survey scores			
Energy expenditure (kcal/min)	73.5 ± 11.4	135.7 ± 30.4	**0.041**
Vigorous activity index	1 ± 1	33 ± 6	** < 0.001**
Leisurely walking index	5 ± 2	23 ± 3	** < 0.001**
Moving index	8 ± 1	9 ± 1	0.466
Standing index	4 ± 1	5 ± 1	0.188
Sitting index	4 ± 0	3 ± 0	**0.041**
Flights of stairs climber per day	2 ± 1	5 ± 2	0.146
Seasonal adjustment score	1 ± 0	1 ± 0	0.437
Mood			
Tension	6 ± 1	5 ± 1	0.290
Depression	8 ± 3	4 ± 2	0.806
Anger	6 ± 2	5 ± 1	0.992
Fatigue	7 ± 2	5 ± 1	0.518
Confusion	7 ± 2	6 ± 1	0.394
Vigour	14 ± 2	20 ± 2	**0.037**
Total mood disturbance	20 ± 10	5 ± 6	0.157

Values are means ± SEM

Results in bold highlight a significant *P* value

### Experimental design

The study utilised a cross-sectional design. Each participant visited the laboratory on two separate occasions, at a similar time of day, separated by a minimum of 24 h and a maximum of 7 days. Participants fasted and abstained from caffeinated stimulants for 1 h before visit 1 and 8–12 h before visit 2. They were also requested to refrain from moderate-vigorous intensity exercise for 24 h before each visit and take their daily supplements and medication after each visit was completed. During visit 1, participants undertook anthropometric, cardiovascular, exercise performance, strength, cerebrovascular and cognitive measurements. At their second visit, participants undertook body composition measurements, blood collection and the Profile of Moods State questionnaire. The Profile of Mood States questionnaire calculated mood disturbance by adding the scores of the negative mood state scales (i.e. anger-hostility, confusion-bewilderment, depression-dejection, fatigue inertia, tension-anxiety) and subtracting the positive mood state scale (i.e. vigour-activity) (Heuchert and McNair 2012). Lower values in the negative mood states and total mood disturbance indicates better mood, while higher values in vigour is associated with positive mood. Between visits, participants were asked to complete a nutritional questionnaire (Automated Self-Administered 24-h Dietary Assessment Tool; National Institute of Health (NIH), Bethesda, MA, USA) to assess energy intake over a typical 24 h period (Pannucci et al. 2018).

### Basal cerebral haemodynamics

Transcranial Doppler ultrasonography (TCD; DopplerBox X; Compumedics DWL, Singen, Germany) was used to measure basal cerebrovascular haemodynamics, including baseline, peak and mean values for both CBF_V_ and cerebral pulsatility, as well as CVR in response to hypercapnia and cognitive stimuli (Barbour et al. 2017; Evans et al. 2017a; Edmonds Jr et al. 2011). Participants were seated and fitted with a headpiece which housed two 2-MHz TCD ultrasound probes that were fixed and aligned bilaterally to the left and right cranial temporal bone windows to insonate the MCA at a depth of approximately 40–65 mm. Once a suitable blood flow signal was obtained, participants were asked to remain seated for at least 10 min before hypercapnia testing. Following the 10-min period, participants were asked to sit quietly while basal measurements were recorded for 30 s.

### Cerebrovascular responsiveness to hypercapnia

Participants were subsequently challenged with a hypercapnic stimulus for 3 min, as a plateau in CBF_V_ is obtained within this time and monitored for another 1 min following removal of this. This process was performed in duplicate following a rest period of at least 5 min (whilst participants breathed in room air in a seated position) to ensure CBF_V_ returned to baseline values (Barbour et al. 2017; Evans et al. 2017a). Participants breathed through a two-way non-rebreathing valve (model 2730, Hans Rudolph, Kansas City, MO, USA) whilst wearing a nose-clip. The inspiratory port of the two-way valve was connected to 1 m of wide bore tubing distal to a 100-L Douglas bag which contained carbogen gas (5% carbon dioxide and 95% oxygen; Carbogen 5; BOC, Toowoomba, QLD, Australia). Flow was measured from the expiratory port of the two-way valve using a pneumotachograph (MLT 300L; AD Instruments, Bella Vista, NSW, Australia) which was calibrated with a 3-L syringe prior to the commencement of each test. Volume was obtained by numerical integration of the flow signal. End-tidal partial pressures of carbon dioxide (P_ET_CO_2_) were sampled from the expiratory port of the two-way valve connected to a gas analyser (ADI ML206; AD Instruments) that was calibrated across the physiological range with known gas concentrations (BOC). Flow and P_ET_CO_2_ measurements were sampled at 200 Hz using a 4-channel Powerlab analog-to-digital converter (AD Instruments) interfaced with a computer and displayed in real time during testing. Data were stored for subsequent offline analysis using LabChart software (version 7.2, AD Instruments).

### Cognitive function and cerebrovascular responsiveness to cognitive stimuli

The individual cognitive tests that comprised the cognitive battery used to assess neuropsychological function included the Trail Making Task Parts A and B which assessed central executive function, Spatial Span Test (visuospatial short-term working memory) and a NIH Toolbox, which is a battery of cognitive examinations (Evans et al. 2017a; Strauss et al. 2006). The NIH Toolbox is comprised of individuals tests and included the Dimensional Change Card Sort Test (cognitive flexibility and attention); Picture Vocabulary Test (language and crystallised cognition); List Sorting Working Memory Test (working memory); Oral Reading Recognition Test (language and crystallised cognition); Flanker Inhibitory Control and Attention Test (attention and inhibitory control); Picture Sequence Memory Test (episodic memory); and Pattern Comparison Processing Speed Test (processing speed) (Heaton et al. 2014; Slotkin et al. 2012). Additionally, a total composite cognitive function score, which was adjusted and controlled for age, sex, education and ethnicity and derived from all of the tests that comprise both fluid and crystallised measurements indicated above, was determined (Heaton et al. 2014). This is a validated and highly reliable score that represents an overall summation of general cognitive function and indicates general cognitive ability based on normalised scores (Heaton et al. 2014; Weintraub et al. 2014; Slotkin et al. 2012). All tests excluding the Trail Making Task were delivered using an iPad (6^th^ generation, Apple Inc, Cupertino, CA, USA). All NIH Toolbox test scores were automatically computed within the program to control for examiner bias. The outputs for all tests were normalised based on the demographics entered into the program (age, education level, familial education history, sex, ethnicity and occupation). A full description of how these tests are administered, how these scores are calculated and the validation of these tests and scores has been previously described in detail (Heaton et al. 2014; Slotkin et al. 2012; Weintraub et al. 2014). These individual cognitive tests were used for the CVR to cognitive stimuli. The CVR to cognitive stimuli was assessed individually for each cognitive task. Thirty seconds of baseline data were recorded before the start of each cognitive task. The total composite (overall) CVR to all cognitive stimuli was summated and averaged based on the number of tests completed. All participants had the same duration of cognitive stimuli applied – i.e. there were no differences in the cognitive tasks administered to the participants of the study.

### Data capture and processing for cerebrovascular responsiveness

Beat-to-beat measurements of CBF_V_ were recorded from the MCA onto software (QL Reader; Compumedics DWL, Singen, Germany) sampling at 100 Hz and were stored for subsequent offline analysis. If a bilateral signal was not obtained, then analysis took place with only the side that was able to be obtained. These data were then normalised and analysed using Curve Expert Professional software (Hyams Development, Chattanooga, TE, USA) to determine resting cerebral pulsatility index (CPI), resting CBF_V_ and peak CBF_V_. CPI and CVR were calculated based on the equations [1] and [2] from previous work (Harris et al. 2018; Wong et al. 2019; Evans et al. 2017b; Nealon et al. 2017; Wijnhoud et al. 2011)

1 $$\mathrm{CPI}= \frac{\mathrm{peak\;systolic\;CBFv}-\mathrm{ end\;diastolic\;CBFv}}{\mathrm{mean\;CBFv\;during\;a\;cardiac\;cycle}}$$

2 $$\mathrm{CVR }(\mathrm{\%})=\frac{\left(\mathrm{peak\;CBFv}-\mathrm{resting\;CBFv}\right)}{\mathrm{resting\;CBFv}}/\mathrm{resting\;CPI}\times 100$$

### Anthropometrics and body composition

Participants were instructed to wear light clothing prior to testing and subsequently asked to remove their shoes for measurements. Body mass was measured to the nearest 100 g using an electronic scale (Tanita Ultimate Scale 2000; Tanita, Tokyo, Japan) and waist and hip circumferences recorded to the nearest 1 cm using a standard tape measure as previously described (Welborn et al. 2003). Height was recorded to the nearest 1 cm using a wall-mounted telescopic stadiometer (Seca220; Vogel & Halke, Hamburg, Germany). Height, body mass and waist and hip circumference measurements were measured in duplicate and the mean of the two measurements were analysed. Body mass index (BMI) and a waist-to-hip ratio were calculated as previously described (Welborn et al. 2003; Keys et al. 1972). Dual-energy X-ray absorptiometry was measured to determine body composition of total lean mass, body fat percentage, and whole-body bone mineral content and density (Luna Corp Prodigy Advance Model GE; Madison, WI, USA).

### Cardiovascular function

Systolic and diastolic blood pressure, mean arterial pressure and arterial elasticity were measured non-invasively using a HDI/Pulsewave™ CR-2000 Research Cardiovascular Profiling System (Hypertension Diagnostics, Eagan, MN, USA) (Prisant et al. 2002). Participants rested in a seated position for 10 min prior to measurements and four consecutive readings were recorded approximately 5 min apart. An automated oscillometer and an appropriately size blood pressure cuff over the left brachial artery were used to assess blood pressure and a tonometer, placed over the right radial artery, to assess pulse wave analysis, heart rate and estimated cardiac output and cardiac index (Barbour et al. 2017; Prisant et al. 2002). The first reading was discarded, and the mean of the three subsequent measurements were used for analysis.

### Biochemical analyses

Approximately 20 ml of venous blood was sampled using a suitable method (either evacuated tube system or winged-infusion) from the veins of the antecubital fossa into thrombin-based clot activator serum separator tubes, 17 IU/ml lithium heparin tubes, 3.2% citrate tubes and 1.8 mg/ml K2 ethylenediaminetetraacetate tubes (BD, Macquarie Park, NSW, Australia). Following collection, blood was either left to stand for 30 min at 18–25 °C (serum separator tube) prior to centrifugation at 1300*g* and 18 °C for 10 min, or centrifuged immediately at 1300*g* and 18 °C for 10 min (plasma tubes) as outlined by the tube manufacturer and the testing laboratory (QML 2019; BD 2019). Following centrifugation, blood was either separated as serum or plasma pending the type of tube used to collect the blood. Samples used for the general chemistry profile and high-sensitivity C-reactive protein (hs-CRP) measurements were performed on a Siemens ADVIA® Labcell® (Siemens Healthcare, Bayswater, VIC, Australia), which utilises spectrophotometric (enzymes, metabolites, proteins, lipids), turbidimetric (hs-CRP) and potentiometric (electrolytes) techniques (Healthineers 2019). The remainder of the serum and plasma was stored at -80 °C for subsequent analyses of vascular endothelial growth factor, which was measured in duplicate using an enzyme-linked immunosorbent assay according to the manufacturer’s instructions (Catalogue No. KHG0111; Invitrogen, Human VEGF ELISA Kit, Vienna, Austria).

### Exercise performance and handgrip strength

Exercise performance was assessed using a 6-min walk test (6MWT) according to published guidelines (ATS 2002). Handgrip strength was determined using hand dynamometry (Jamar Digital Plus; Lafayette Instruments, Lafayette, IN, USA) with an adequate rest period of 1 min between each attempt to limit fatigue, as previously described (Hillman et al. 2005). Participants were permitted three attempts with both their dominant and non-dominant hands. The first reading for each hand was discarded and was used as a familiarisation and the second and third readings for each hand were averaged for each hand and were used for analysis.

### Statistical analysis

Statistical analyses were performed using SPSS for Windows (IBM, Chicago, IL, USA). An initial power calculation was performed on the basis of previous research that has investigated the differences in CVR between population groups (Barbour et al. 2017; Evans et al. 2017a; Wong et al. 2016a, c). The power analysis demonstrated that a sample size of 12 per group would be required to detect a 5% difference in CVR between trained and untrained participants (alpha = 0.05 and power = 0.8), which has been previously shown to be positively correlated with statistically significant improvements in cognition (Barbour et al. 2017). Normality of data was assessed using a Shapiro–Wilk test. Comparisons between groups for anthropometric, body composition, cardiovascular, cognitive, exercise performance, baseline cerebrovascular, baseline respiratory, both CVR to hypercapnia and CVR to cognitive stimuli, strength and biochemical measures were determined using independent *t* test or Mann–Whitney U-tests for parametric and non-parametric data, respectively. Between-group differences for raw cerebrovascular (excluding CVR) and respiratory measures were analysed using a two-way analysis of variance to determine the effects of ‘group’ (trained vs. untrained) and ‘time’ (baseline vs. peak during hypercapnia). Significant group × time interaction effects were followed by planned pairwise comparisons between groups using the Bonferroni method. Pearson’s product moment correlation coefficient (parametric data) or Spearman’s (non-parametric data) correlation analysis was used to examine the relationship between variables and reported cut-off points to examine these relationships were applied as previously described (Schober et al. 2018). An analysis of co-variance (ANCOVA) was performed using objective (non-self-reported) measures that demonstrated a significant relationship with the primary outcomes (covariates) as independent variables and the primary outcomes (CVR to hypercapnia; CVR to cognitive stimuli; total composite cognitive score) as dependent variables. These objective measures included years of education, 6MWT distance, large arterial compliance, serum hs-CRP concentration, waist circumference, total body fat percentage, heart rate and breathing frequency. Additionally, one measure of total body composition (total body fat percentage) and only biochemical measurements that were considered clinically significant (i.e. were outside reference ranges) were used in the ANCOVA if significant in the correlation analyses. Statistical significance was set at *P* < 0.05. Data are presented as means ± SEM.

## Results

### Participant characteristics

Participant characteristics are shown in Table 1. The types of aerobic exercise types that participants undertook, including duration in years, are shown in Supplemental Table 1.There were no differences between the groups for age, sex and height. The trained group had spent more time in education compared to the untrained group. The trained group had a lower body mass, hip circumference, waist circumference, total body fat and hip-to-waist ratio than the untrained group. The trained group walked for longer during the 6MWT and had a higher total energy output, sitting index score and vigorous activity and leisurely walking index score than the untrained group. The trained group had a higher vigour score than the untrained group. There were no other differences in participant characteristics between the groups.

### Biochemical analyses

Participants’ general biochemistry profiles, hs-CRP and vascular endothelial growth factor are shown in Table 2. Serum sodium and high-density lipoprotein concentrations were higher in the trained compared to the untrained group. Serum glucose, triglycerides, hs-CRP, alkaline phosphatase and alanine aminotransferase concentrations were lower in the trained compared to the untrained group. There were no other differences in the general chemistry profile and vascular endothelial growth factor between the groups.

**Table 2:** Biochemical analyses for the untrained and trained groups. Values are means ± SEM

Variable	Untrained (*n* = 13)	Trained (*n* = 12)	*P* value
Sodium (mmol/L)	139 ± 1	142 ± 1	**0.006**
Potassium (mmol/L)	4.7 ± 0.1	4.4 ± 0.1	0.078
Chloride (mmol/L)	105 ± 1	106 ± 1	0.206
Bicarbonate (mmol/L)	25 ± 1	25 ± 1	0.405
Glucose (mmol/L)	5.8 ± 0.4	4.9 ± 0.2	**0.022**
Urea (mmol/L)	5.9 ± 0.4	6.3 ± 0.7	0.910
Creatinine (mmol/L)	77 ± 5	77 ± 4	0.971
Estimated glomerular filtration rate (ml/min)	80 ± 4	79 ± 3	0.364
Urate (mmol/L)	0.34 ± 0.01	0.32 ± 0.02	0.354
Total bilirubin (µmol/L)	10 ± 1	12 ± 1	0.111
Alkaline phosphatase (U/L)	91 ± 7	63 ± 6	**0.008**
Gamma-glutamyl transferase (U/L)	37 ± 6	27 ± 4	0.190
Alanine aminotransferase (U/L)	36 ± 5	24 ± 4	**0.003**
Aspartate aminotransferase (U/L)	33 ± 4	27 ± 3	0.258
Lactate dehydrogenase (U/L)	216 ± 16	216 ± 17	0.870
Calcium (mmol/L)	2.32 ± 0.02	2.36 ± 0.04	0.172
Corrected calcium (mmol/L)	2.38 ± 0.03	2.37 ± 0.03	0.587
Phosphate (mmol/L)	1.1 ± 0.0	1.2 ± 0.0	0.271
Total protein (g/L)	69 ± 1	69 ± 1	0.877
Albumin (g/L)	41 ± 1	42 ± 1	0.601
Globulins (g/L)	28 ± 1	27 ± 1	0.440
Total cholesterol (mmol/L)	5.2 ± 0.3	5.3 ± 0.2	0.329
Triglycerides (mmol/L)	1.7 ± 0.2	1.1 ± 0.1	**0.042**
High-density lipoprotein (mmol/L)	1.37 ± 0.08	1.63 ± 0.10	**0.047**
Low-density lipoprotein (mmol/L)	2.83 ± 0.24	2.86 ± 0.19	0.970
Total cholesterol-to-high-density lipoprotein ratio	3.9 ± 0.3	3.4 ± 0.2	0.139
High-sensitivity C-reactive protein (mg/L)	4.3 ± 1.0	1.0 ± 0.4	**0.002**
Vascular endothelial growth factor (pg/mL)	273.3 ± 42.5	205.8 ± 34.3	0.241

Results in bold highlight a significant *P* value

### Cardiovascular function

Cardiovascular function is shown in Table 3. Heart rate, cardiac output, and systolic, diastolic and mean arterial blood pressure were lower in the trained group compared to the untrained group. Large arterial compliance was higher in the trained group compared to the untrained group. There were no other differences in cardiovascular function between the groups.

**Table 3:** Cardiovascular function for the untrained and trained groups. Values are means ± SEM

Variable	Untrained (*n* = 13)	Trained (*n* = 13)	*P* value
Heart rate (beats/min)	76 ± 3	56 ± 2	** < 0.001**
Cardiac output (L/min)	5.3 ± 0.3	4.4 ± 0.2	**0.024**
Cardiac index (L/min/m^2^)	2.5 ± 0.1	2.5 ± 0.1	0.583
Systolic blood pressure (mmHg)	143 ± 3	126 ± 4	**0.003**
Diastolic blood pressure (mmHg)	78 ± 2	69 ± 2	**0.014**
Mean arterial pressure (mmHg)	104 ± 3	93 ± 2	**0.009**
Large arterial compliance (ml/mmHg × 10)	9.3 ± 1.0	13.6 ± 1.1	**0.008**
Small arterial compliance (ml/mmHg × 10)	4.7 ± 0.6	3.9 ± 0.4	0.659
Systemic vascular resistance (dyne/s/cm^−s^)	1560 ± 75	1710 ± 85	0.200
Total vascular impedance (dyne/s/cm^−s^)	180 ± 12	178 ± 12	0.911

Results in bold highlight a significant *P* value

### Cerebrovascular responsiveness to hypercapnia

**Fig. 1:**
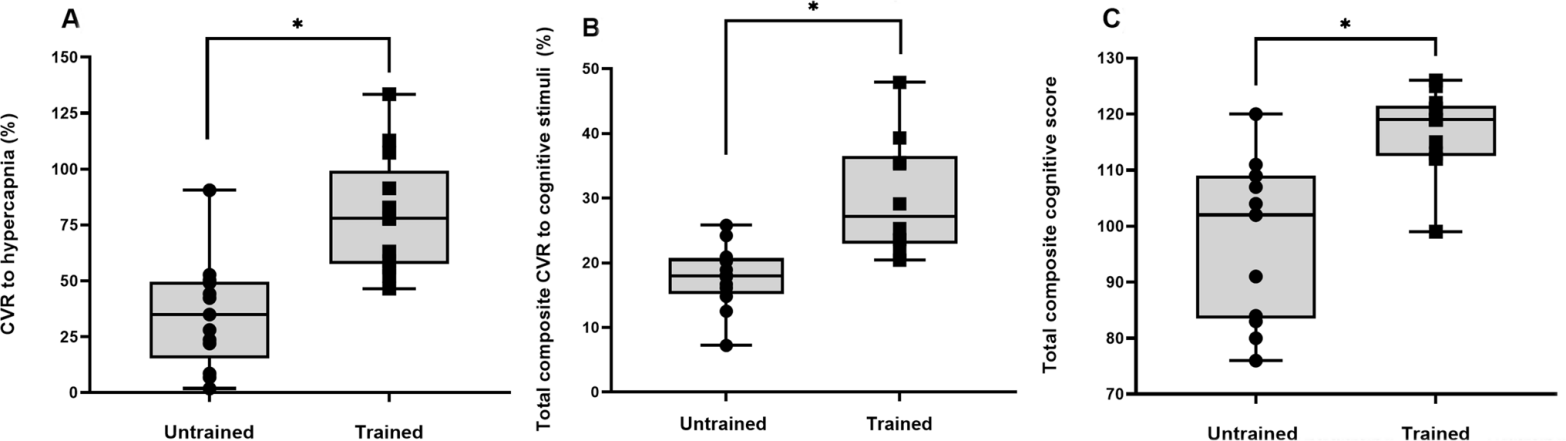
Cerebrovascular responsiveness (CVR) to hypercapnia (**A**), CVR to total composite of cognitive stimuli (**B**) and total composite cognitive score (**C**) for untrained (*n* = 13) and trained (*n* = 13) groups. *Significant difference between groups (*P* < 0.001)

The CVR to hypercapnia is shown in Fig. 1 and Table 4. There were no differences in TCD signal laterality between the groups (untrained unilateral signal, *n* = 4 vs. trained unilateral signal, *n* = 5; *P* = 0.695). There were no differences in CBF_V_ at baseline. The CVR to hypercapnia (53% higher) and CPI were higher for the trained than the untrained group*.* There was an increase (main effect of time) in CBF_V_, CPI, P_ET_CO_2_ and tidal volume in response to hypercapnia. The responses (time × group interaction) of CBF_V_ and CPI were also higher for the trained than the untrained group. Breathing frequency was lower for the trained compared to the untrained group, but there were no time × group interactions.

**Table 4:** Cerebrovascular responsiveness to hypercapnia for the untrained and trained groups. Values are means ± SEM

Variable	Untrained (*n* = 13)		Trained (*n* = 13)		*P* value		
	Baseline	Peak	Baseline	Peak	Time	Group	Time × Group
CBF_V_ (cm/s)	33.1 ± 2.8	45.4 ± 4.0	36.5 ± 2.9	60.1 ± 4.0	** < 0.001**	0.068	** < 0.001**
CBF_V_/P_ET_CO_2_ (cm/s/mmHg)	1.2 ± 0.1	1.5 ± 0.1	1.1 ± 0.1	1.6 ± 0.1	** < 0.001**	0.795	0.065
Cerebral pulsatility index	1.28 ± 0.08	0.95 ± 0.05	0.91 ± 0.03	0.80 ± 0.03	** < 0.001**	**0.001**	**0.004**
P_ET_CO_2_ (mmHg)	30.7 ± 2.2	35.1 ± 1.9	32.4 ± 0.9	38.3 ± 0.9	** < 0.001**	0.207	0.191
Tidal volume (L)	0.69 ± 0.09	0.95 ± 0.12	1.11 ± 0.19	1.40 ± 0.19	** < 0.001**	0.080	0.697
Breathing frequency (breaths/min)	19 ± 2	20 ± 3	11 ± 1	11 ± 1	0.645	**0.001**	0.579
Minute ventilation (L/min)	12.9 ± 1.7	17.6 ± 3.2	12.5 ± 2.5	13.1 ± 1.7	0.065	0.430	0.157

Results in bold highlight a significant *P* value

*CBF*_*V*_ cerebral blood flow velocity, *P*_ET_*CO*_2_ partial pressure of end tidal carbon dioxide

### Cognitive function and cerebrovascular responsiveness to cognitive stimuli

Cognitive function and cerebrovascular responses to cognitive stimuli are shown in Fig. 1 and Table 5. The trained group had higher overall cognitive function than the untrained group, which was demonstrated by a higher total cognitive composite score (17% higher). The trained group also had higher cognitive function than the untrained group in the Dimensional Change Card Sort Test (cognitive flexibility and attention), the List Sorting Working Memory Test (working memory), and Oral Reading Recognition Test (crystallised cognition). The trained group completed both sections of the Trail Making Task (executive function) in less time with fewer errors made in Part B of the task than the untrained group. The time difference between Parts B and A of the Trail Making Task was lower in the trained compared to the untrained group.

**Table 5:** Cognitive function and cerebrovascular responsiveness to cognitive stimuli for the untrained and trained groups. Values are means ± SEM

Variable	Untrained (*n* = 13)	Trained (*n* = 13)	*P* value
Cognitive function			
Dimensional change card sort^a^	7.26 ± 0.57	8.46 ± 0.15	**0.048**
Pattern comparison processing speed^a^	43 ± 3	50 ± 3	0.220
Picture vocabulary test^a^	5.77 ± 0.65	6.62 ± 0.52	0.149
Flanker inhibitory control and attention^a^	7.89 ± 0.15	8.12 ± 0.11	0.317
Picture sequence memory^a^	− 0.93 ± 0.25	− 0.41 ± 0.24	0.136
List sorting working memory^a^	15 ± 1	20 ± 0	** < 0.001**
Oral reading recognition^a^	4.17 ± 0.80	8.12 ± 0.34	**0.001**
Trail making task (Part A)			
Time (s)	38.4 ± 3.9	27.0 ± 1.6	**0.044**
Errors made	0.8 ± 0.3	0.2 ± 0.1	0.081
Trail making task (Part B)			
Time (s)	89.9 ± 15.1	48.0 ± 3.4	**0.001**
Errors made	2.6 ± 0.6	0.6 ± 0.4	**0.020**
Part B – Part A time difference	51.5 ± 11.7	21.0 ± 3.9	**0.006**
Spatial Span Test			
Time (s)	104 ± 8	116 ± 6	0.277
Total spans completed	5.1 ± 0.4	5.8 ± 0.2	0.186
Stress			
Prior to cognitive testing	1.85 ± 0.38	2.20 ± 0.38	0.605
Post cognitive testing	3.14 ± 0.66	3.14 ± 0.72	0.999
Difference	1.29 ± 0.46	0.93 ± 0.83	0.713
Mental Fatigue			
Prior to cognitive testing	2.53 ± 0.39	2.69 ± 0.59	0.827
Post cognitive testing	3.46 ± 0.54	4.64 ± 0.78	0.223
Difference	0.93 ± 0.52	1.95 ± 0.83	0.308
Cerebrovascular responses to cognitive stimuli (%)			
Dimensional change card sort test	15.8 ± 2.9	26.0 ± 3.1	**0.027**
Pattern comparison processing speed test	19.6 ± 2.2	28.0 ± 3.1	0.085
Picture vocabulary test	20.7 ± 2.7	32.0 ± 3.0	**0.032**
Flanker inhibitory control and attention test	11.9 ± 2.2	25.7 ± 3.1	**0.003**
Picture sequence memory test	19.2 ± 2.3	35.8 ± 4.0	**0.006**
List sorting working memory test	18.7 ± 1.7	32.7 ± 5.3	**0.049**
Oral reading recognition test	15.3 ± 2.0	23.7 ± 3.8	0.146
Trail making task (Part A)	19.8 ± 2.9	38.6 ± 5.9	**0.038**
Trail making task (Part B)	18.5 ± 2.2	31.8 ± 4.1	0.080
Spatial Span Test	17.2 ± 2.2	31.4 ± 3.7	**0.012**

Results in bold highlight a significant *P* value

^a^Normalised, computed and standardised automatically by NIH Toolbox, based on validated measures (Slotkin et al. 2012)

The total composite (overall) CVR to all cognitive stimuli was higher in the trained compared to the untrained group (40% higher). The trained group had a higher CVR to cognitive stimuli compared with the untrained groups during the Dimensional Change Card Sort test, Picture Vocabulary Test, Flanker Inhibitory Control and Attention Test, Picture Sequence Memory Test, List Sorting Working Memory Test, Part A of the Trail Making Task and the Spatial Span Test.

Participants were requested to indicate their perceived level of stress and mental fatigue using a digitised visual analogue scale (Visual Scale; Bit Genoma Digital Solutions SL, Badalona, Spain) pre and post cognitive testing.

### Correlations between measured variables and cerebrovascular responsiveness to hypercapnia and cognitive stimuli, and cognitive function

**Fig. 2:**
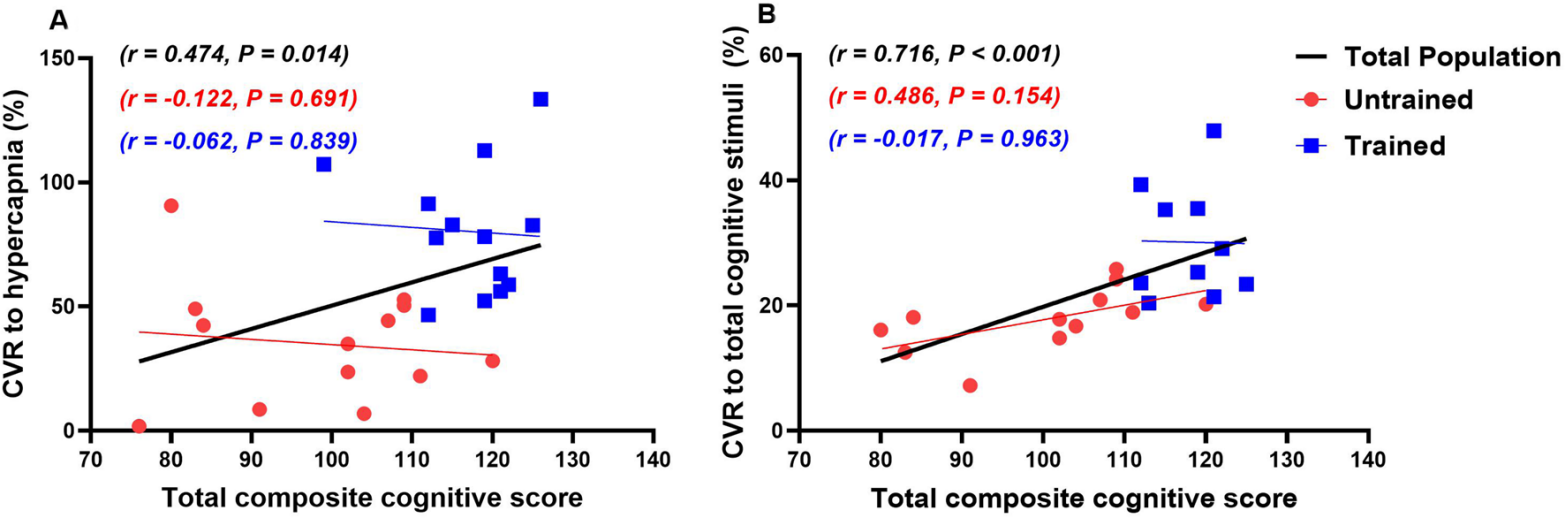
Correlations between cerebrovascular responsiveness (CVR) to hypercapnia (**A**) total composite CVR to cognitive stimuli (**B**) and total composite cognitive score

Significant correlations between measured variables and CVR to hypercapnia and cognitive stimuli, and cognitive function are shown in Table 6 and Fig. 2. There were moderate positive correlations between CVR to hypercapnia and CVR to cognitive stimuli and the total composite cognitive score. There was a strong positive correlation between the total composite CVR to cognitive stimuli and total composite cognitive score. Both CVR to cognitive stimuli and total composite cognitive score were also strongly correlated with consistent exercise training (years).

**Table 6:** Correlations between measured variables and cerebrovascular responsiveness (CVR) to hypercapnia and cognitive stimuli, and cognitive function (total composite cognitive score)

Variable	CVR to hypercapnia		CVR to cognitive stimuli		Cognitive function	
	*r* value	*P* value	*r* value	*P* value	*r* value	*P* value
Education (years)	0.620	0.001	0.590	0.002	0.611	0.003
Consistent exercise training (years)	0.503	0.009	0.643	< 0.001	0.682	< 0.001
Body mass (kg)	− 0.398	0.044	− 0.538	0.005	− 0.456	0.033
Height (m)	− 0.482	0.013	− 0.550	0.004	− 0.477	0.025
Hip circumference (cm)	− 0.568	0.002	− 0.532	0.005	− 0.421	0.051
Waist circumference (cm)	− 0.509	0.008	− 0.631	0.001	− 0.574	0.005
Hip-to-waist circumference ratio	− 0.301	0.135	− 0.538	0.002	− 0.532	0.011
Total body fat (%)	− 0.575	0.003	− 0.396	0.050	− 0.353	0.116
6-min walk test distance (m)	0.483	0.012	0.551	0.004	0.566	0.006
High-sensitivity C-reactive protein (mg/L)	− 0.687	< 0.001	− 0.403	0.051	− 0.241	0.306
Heart rate (beats/min)	− 0.573	0.002	− 0.606	0.001	− 0.533	0.011
Large arterial compliance (ml/mmHg × 10)	0.397	0.045	0.434	0.027	0.321	0.145
Baseline breathing frequency (breaths/min)	− 0.585	0.003	− 0.541	0.008	− 0.561	0.012
Peak breathing frequency (breaths/min)	− 0.400	0.058	− 0.666	0.001	− 0.575	0.010
CVR to hypercapnia (%)	–	–	0.553	0.008	0.474	0.014
CVR to cognitive stimuli (%)	0.553	0.008	–	–	0.685	< 0.001
Total composite cognitive score	0.474	0.014	0.716	< 0.001	–	–

There were moderate positive correlations between CVR to hypercapnia and the total years educated, 6MWT distance and large arterial compliance. There were moderate negative correlations between CVR to hypercapnia and serum hs-CRP concentration, waist circumference, total body fat percentage, heart rate and baseline breathing frequency.

There were moderate positive correlations between the total composite cognitive score and total years educated and, 6MWT distance. There were moderate negative correlations between the total composite cognitive score and peak breathing frequency during hypercapnia, waist circumference and heart rate. There was a moderate negative correlation between the total composite cognitive score and large arterial compliance.

There were moderate positive correlations between the total composite CVR to cognitive stimuli and total years educated and 6MWT distance. There were moderate negative correlations between the total composite CVR to cognitive stimuli and waist circumference, heart rate, and breathing frequency both at baseline and peak breathing frequency during hypercapnia.

### Analysis of covariance between the primary outcomes and covariates

Objective measures that demonstrated a significant relationship with the primary outcomes (CVR to hypercapnia; CVR to cognitive stimuli; total composite cognitive score) and were considered clinically significant were used in the ANCOVA (described above; shown in Table 6). The reference ranges used for determining clinical significance of these values were derived from the Royal College of Pathologists of Australasia, Royal Australian College of General Practitioners, Australian Heart Foundation and the American Thoracic Society (ATS 2002; Harris 2013; RCPA 2021). Those that demonstrated significant relationships with the primary outcomes and were clinically significant included years of education, 6MWT distance, large arterial compliance, serum hs-CRP concentration, waist circumference, total body fat percentage, heart rate and breathing frequency. Following adjustment for covariates (years of education, 6MWT distance, large arterial compliance, serum hs-CRP concentration, waist circumference, total body fat percentage, heart rate and resting breathing frequency) the ANCOVA revealed that the CVR to hypercapnia no longer remained statistically different between the groups (*P* = 0.934). The ANCOVA performed for the composite CVR to cognitive stimuli (covariates: total years educated, 6MWT distance, maximum breathing frequency during hypercapnia and at rest, waist circumference measurement and heart rate; *P* = 0.343) was also not statistically different between the groups. This was the same for the ANCOVA performed for the total composite cognitive score (covariates: total years educated, total composite CVR to cognitive stimuli, 6MWT distance, CVR to hypercapnia, maximum breathing frequency during hypercapnia, waist circumference measurement, heart rate and large arterial compliance; *P* = 0.202). Education level was also strongly correlated with the primary outcomes. When education was removed from the ANCOVA and all other significant variables listed above were left in the analysis, the results were still insignificant (CVR to hypercapnia, *P* = 0.246; CVR to total composite of cognitive stimuli, *P* = 0.193; and total composite cognitive score, *P* = 0.940).

## Discussion

### Main findings

The aims of our study were threefold. First, to compare the differences in cerebrovascular and cognitive function between older adults that had undertaken regular aerobic exercise training over the last 40 years and those that were sedentary and untrained. Second, to determine the association between cerebrovascular and cognitive function in these individuals. Finally, to examine if other measures including body composition, cardiovascular function, biochemistry and educational level would account for the differences between trained and untrained groups. In support of our aims, the main findings were that, first, the trained group had undertaken 40 years of aerobic exercise training and had higher cerebrovascular and cognitive functions than the untrained group. Second, there were moderate positive correlations between CVR to hypercapnia and total composite cognitive score and CVR to cognitive stimuli. Finally, following adjustment for covariates, CVR to hypercapnia, total composite cognitive score and CVR to cognitive stimuli were not significantly different between the groups. This suggests that there are many factors that affect cognition and cerebrovascular function that are associated with physical inactivity, such as increased adiposity, blood pressure and heart rate, and that lifelong aerobic exercise training may mitigate these adverse effects.

### Cerebrovascular responsiveness to hypercapnia

We observed that the CVR to hypercapnia was higher for the trained than the untrained group. This finding supports previous studies that have reported a higher CVR to hypercapnia in older aerobic trained individuals (Bailey et al. 2013; Barnes et al. 2013; Marley et al. 2020; Rogers et al. 1990; Brown et al. 2010), but not others that have reported no differences between trained and untrained groups (Braz et al. 2017; Thomas et al. 2013; Zhu et al. 2013; Miller et al. 2018). We found no differences in resting CBF_V_ between the trained and untrained groups, which contradicted previous studies that have reported a higher CBF_V_ in aerobic exercise trained individuals (Ainslie et al. 2008; Bailey et al. 2013). However, previous research has also reported no differences in resting CBF_V_ following an exercise intervention or between trained and untrained individuals, respectively (Vicente-Campos et al. 2012; Thomas et al. 2013; Braz et al. 2017). The discrepancy between our findings and others is not clear but may be due to the potential variation in aerobic fitness levels between the trained and untrained participants, the types of training others had participated in and using only a single sex for analysis. For example, Ainslie et al. (2008) only recruited men who participated in cycling or running and did not report the final maximum oxygen consumption test. However, Thomas et al. (2013) only recruited competitive and nationally ranked men and women Masters runners whose maximum oxygen consumption was 41 mL/kg/min compared with sedentary individuals who reported a value of 23 mL/kg/min. Braz et al. (2017) recruited men who participated in any type of aerobic exercise as long as they met the definition of being aerobic exercise trained, but reported similar maximum oxygen consumption test values to Thomas in the trained group (40 mL/kg/min), but much higher values in the untrained group (31 mL/kg/min). Additionally, we also observed a higher peak CBF_V_ in the trained compared to the untrained group and a lower CPI both at rest and during hypercapnia, which is consistent with the literature (Bailey et al. 2013; Marley et al. 2020; Barnes et al. 2013; Mohammadi et al. 2021).

The higher CVR to hypercapnia reflects an enhanced ability of the cerebrovasculature to modify regional blood flow in response to local chemical changes (i.e. carbon dioxide) and maintain cerebral autoregulation. The finding may suggest that regular aerobic exercise training can improve the vasomotor reactivity of the blood vessels and reduce arterial stiffness within the cerebrovasculature (Rogers et al. 1985; Miller et al. 2018). Furthermore, aerobic exercise training could be related to favourable changes in cardiovascular structure and function systemically and this is transferrable centrally (Bailey et al. 2013). For example, it was observed that the trained group had lower central adiposity, blood triglycerides and blood glucose, as well as higher blood high-density lipoprotein concentrations and cardiovascular parameters. Additionally, hs-CRP, which is a marker of chronic low-grade systemic inflammation and predictor of risk of an acute cardiovascular or cerebrovascular event (Jialal and Devaraj 2001), was lower in the trained group. The differences in these parameters between the groups are consistent with the benefits associated with consistent aerobic exercise training (Rossman et al. 2018; Lin et al. 2015). These results also highlight the poor cardiometabolic status of the untrained group and this may place them at greater risk of suffering from both decreased cerebrovascular function and cognitive decline (Toth et al. 2017; Woods et al. 2011; Bliss et al. 2021). The higher concentrations of hs-CRP and metabolic markers, such as blood glucose and triglycerides, in the untrained group may also be an indication of reduced endothelial function, which was observed in this study, thereby promoting increased arterial stiffness, thus reducing blood flow and vasoreactivity (Toth et al. 2017; Woods et al. 2011; Bangen et al. 2014; Toda 2012). The reduction in cerebrovascular function, in turn, reduces the metabolic capacity of the brain, as the brain is no longer supplied as efficiently as it once was with essential nutrients, oxygen and its metabolic waste is no longer removed as quickly as in earlier life (Toth et al. 2017; Bliss et al. 2021; Bangen et al. 2014). Therefore, our results support the notion that aerobic exercise training may modulate these changes, preventing or slowing down these processes that are also associated with ageing.

### Cognitive function and cerebrovascular responsiveness to cognitive stimuli

We observed that the total composite cognitive score was higher in the trained compared to the untrained group. While we also evaluated specific cognitive measurements that utilise different cognitive domains, the total composite score evaluates a variety of cognitive domains that are used during activities of daily living (Harvey 2019). Each cognitive domain and subtype can directly impact one another (i.e. they are interdependent) (Harvey 2019; Salthouse 2012). Diseases associated with cognitive decline and neurodegeneration typically do not affect a single domain either, rather they impact all cognitive domains (Harvey 2019; Salthouse 2012). The differences in cognitive function between the groups support the current literature, which indicates that regular aerobic exercise training improves cognitive function (Anderson-Hanley et al. 2012; Baker et al. 2010; Bossers et al. 2015; Hoffmann et al. 2016; Lautenschlager et al. 2008; Sobol et al. 2016; Vreugdenhil et al. 2012). The mechanisms for how aerobic exercise training improves cognitive function in humans is poorly understood. Existing knowledge on this mechanism has been mainly drawn from animals studies (Bliss et al. 2021). Improvements in cognition may be attributed to increased brain-derived neurotrophic factor (BDNF) concentrations both systemically and centrally. BDNF acts to promotes synaptogenesis and has been associated with increased hippocampal volume and spatial memory (Erickson et al. 2011) in humans and improved cognition in animal studies (Vaynman et al. 2004). However, the exact role of BDNF in improving neuroplasticity and cognition in different disease states, such as Parkinson’s disease, still remains inconclusive (Johansson et al. 2020).

We also observed that the total composite CVR to cognitive stimuli, which is referred to as NVC, was higher in the trained compared with the untrained group. NVC is the complex interaction between neuronal metabolic demands and local haemodynamic changes, which ensures that metabolic demands are met by the vasculature (Duchemin et al. 2012; Toth et al. 2017). The mechanisms that lead to the regulation of CBF, which allow for the prevention of both ischaemia and hyperaemia at any time, are extremely complex and yet to be fully elucidated. In any case, it is known that the upregulation of endothelial nitric oxide synthase and the subsequent synthesis and release of nitric oxide from the endothelium is a fundamental component involved in both cerebrovascular autoregulation and NVC (Duchemin et al. 2012; Toth et al. 2017). Only one other study that has measured NVC in older trained and untrained individuals. Fabiani et al. (2014) reported that NVC varied according to aerobic fitness level and that individuals with greater aerobic fitness had a higher NVC capacity. However, the authors of this study did not measure the CVR to cognitive stimuli, rather to visual stimuli. Hence, our study is the first study to specifically measure the CVR to individual cognitive tasks, as well as cumulatively. Further, our study is the first to determine an association between cerebrovascular and cognitive function in this particular cohort. The authors of this study suggested that the difference in the NVC responses between trained and untrained individuals were most probably associated with the cardiovascular system and noted that older participants with low aerobic fitness were also hypertensive and overweight or obese. This is similar to our findings in that the untrained participants met the clinical criteria for obesity as defined by BMI and waist circumference (Harris 2013), which was validated by observing a significantly increased total body fat percentage using dual-energy X-ray absorptiometry. We also observed differences between the groups in markers of cardiometabolic health (i.e. body composition, cardiovascular function and biochemical analyses, such as hs-CRP, glucose and lipid profile). Since ageing and cardiometabolic health both directly impact endothelial function, promote arterial stiffness and chronic low-grade systemic inflammation, it would be expected that anything that favours the senescent phenotype or exacerbates endothelial dysfunction, such as obesity, would directly impact NVC and, in turn, cognition.

### Correlations between cerebrovascular and cognitive function

The potential association between cerebrovascular function cognitive function is supported by our finding that total composite cognitive scores were moderately positively correlated with CVR to hypercapnia and strongly positively correlated with CVR to cognitive stimuli. Correlation studies between cerebrovascular function and cognition in trained individuals have only been reported once, in which cerebrovascular function was shown to be a positive predictor of overall cognition in older women (Brown et al. 2010). However, other studies have also reported a correlation between cerebrovascular and cognitive function in healthy post-menopausal women (Wong et al. 2016b), hypertensive older adults (Hajjar et al. 2014), Alzheimer’s disease (Richiardi et al. 2015), and healthy older adults (Keage et al. 2015). It is likely that hypertensive older adults and those with Alzheimer’s disease are sedentary. Therefore, it is difficult to make a conclusion based on our findings on the regular aerobic training-cerebrovascular function-cognition relationship. Further studies will need to delineate these relationships by incorporating exercise status into their studies.

The association between cognitive performance and CVR to hypercapnia may be modest as the latter measures a generalised response of the cerebrovasculature to a chemical stimulus and reflects the ability of the cerebrovasculature to respond solely to this stimulus (e.g. increased concentrations of carbon dioxide) (Barnes et al. 2013; Bailey et al. 2013). This is in contrast to NVC, which describes the mechanical response of the neurovascular unit during localised neuronal activation and exertion (i.e. response to increased neuronal metabolism and signalling) (Zlokovic 2011). Hence, while both parameters may be important to holistically determine cerebrovascular function, it is NVC that is most probably specifically related to cognitive function as potentially indicated by the results of this study (Bliss et al. 2021; Toth et al. 2017; Zlokovic 2011). Further, decreased endothelial function and reduced NVC affect cognition, simply because if the haemodynamic response is blunted then there is insufficient essential nutrients and oxygen being made available to supply neurons during times of increased metabolism (Bliss et al. 2021). This essentially affects the ability of the neurons to perform work efficiently and effectively, thus contributing to decreased cognitive function, which, over time, dissipates further leading to cognitive decline.

### Analysis of covariance between the primary outcomes and covariates

When we adjusted for other covariates including body composition, cardiovascular function, biochemistry and educational level that may also explain the differences in cerebrovascular and cognitive function between the trained and untrained groups, the difference between them became statistically insignificant. This finding may suggest that exercise probably modifies multiple physiological variables which have an impact on and interaction with cognitive and cerebrovascular function. It also potentially suggests that having a favourable cardiometabolic profile, which is imperative to maintaining endothelial function, may assist in maintaining the autoregulatory function of the cerebrovasculature throughout the lifespan. The finding may also indicate that exercise could positively contribute to this status by reducing central adiposity, reducing low-grade chronic systemic inflammation, improving cardiovascular structure and function and thereby potentially improving cerebrovascular function and cognition. However, no long-term follow-up studies have been performed to confirm whether improvement in cerebrovascular function will directly improve cognition, but based on empirical studies, the relationship appears to be quite clear (Bliss et al. 2021).

In support of our findings are the results of our correlation analyses. Some of the strongest associations made between our primary outcomes (CVR to hypercapnia, CVR to cognitive stimuli and cognitive function) were with exercise (years consistently performed exercise training and 6MWT distance). Other associations were made between cardiorespiratory variables, central adiposity and low-grade systemic inflammation. Aerobic exercise training reduces adiposity and the chronic low-grade systemic inflammation that is associated with increased central adiposity (You et al. 2013; Keating et al. 2015). Additionally, reductions in cerebrovascular function and structure are largely attributed to modifiable risks factors that impact on cardiometabolic health, particularly physical inactivity (Ahtiluoto et al. 2010; Anstey et al. 2014; Bunch et al. 2010; Kalmijn et al. 2002; Kokmen et al. 1996; Kuller et al. 2005; Prins et al. 2002; Sabia et al. 2018; Scarmeas et al. 2009; Seliger et al. 2004; Solomon et al. 2009). Physical inactivity, in conjunction with reduced cardiometabolic health, promotes and exacerbates the development of chronic low-grade systemic inflammation and reduced endothelial function (Toda 2012; Woods et al. 2011; Bangen et al. 2014; Toth et al. 2017; AIHW 2012). Since physical inactivity promotes increased adiposity and low-grade systemic inflammation, which, in turn, can drive reactive oxygen species production thus reducing endothelial function, it may further exacerbate and reduce cerebrovascular function due to a reduction in capillary density and increased arterial stiffness (i.e. reduced blood flow and vasoreactivity) (Bangen et al. 2014; Toth et al. 2017; Toda 2012; Woods et al. 2011).

Finally, the only other variable that was strongly correlated with the primary outcomes was education. When education was removed as a covariate from the ANCOVA and all other variables mentioned used in this modelling (described above) were used as covariates (i.e. when we just removed education from the analysis), the results were still not significant between the groups. Currently, there is limited work, if any, that has determined the effects of education level on cerebrovascular function other than in Alzheimer’s disease, where it has been reported that higher regional CBF is associated with higher levels of education in these individuals (Chiu et al. 2004). However, more studies are required to determine the effect of education level on cerebrovascular function. Education duration has been correlated to increased cognitive capacity throughout the lifespan and as a potential attenuator of cognitive decline associated with ageing (Lövdén et al. 2020). Therefore, the impact of this on cognitive outcomes in this study cannot be excluded, even though an individual’s level of education is considered by the NIH Toolbox’s scoring algorithm (i.e. education is controlled for by the NIH Toolbox) (Heaton et al. 2014; Weintraub et al. 2014; Slotkin et al. 2012). Educational differences may also be an inherent complication with recruiting individuals for human trials, as the compliance and motivation to participate in studies appears to be higher in those who are more educated compared with those that are not (Mbuagbaw et al. 2017). This may pose a challenge to control for not only in this study but others as well, as others have highlighted education as an important that may affect clinical trials associated with neurodegenerative diseases, such as dementia (Huang et al. 2020). Interestingly, those with higher education levels also appear to progress more rapidly through the course of Alzheimer’s disease compared with those with lower educational levels (Kemppainen et al. 2008), meaning that the relationship of education and cognitive decline, particularly in relation to dementias, still requires more investigation. In any case, our results suggest that, while education is a significant element in maintaining overall brain health, having a favourable cardiometabolic profile is just as important in maintaining both cerebrovascular and cognitive function and that all of these elements are probably inter-related when it comes to improving or maintaining overall brain health throughout the lifespan.

### Methodological limitations

Different techniques have been used to assess cerebrovascular function, such as direct methods like magnetic resonance imaging (MRI) and indirect methods such as TCD (see (Joris et al. 2018) for detailed comparison). CVR is a quantitative measure of the potential of the cerebral microcirculation to dilate in response to physical or chemical stimuli and is a surrogate measure of local endothelial function (Joris et al. 2018; Rossman et al. 2018). It is not a direct method in determining CBF differences in specific regions of the brain in response to a stimulus such as a cognitive task. We acknowledge that CVR is a crude measure that records changes essentially in one vessel only, which in this case is the MCA. We chose to use the MCA as changes in its blood flow velocity more directly reflect changes in CBF arising from the dilatation of the downstream microvasculature supplying brain regions that are critical for cognitive function (Braz et al. 2017; Serrador et al. 2000; Willie et al. 2011). Certain responses to different tests may not have been captured using TCD and by not excluding participants on the basis laterality. TCD and the use of the MCA only may not explain the magnitude differences in one test compared to another in specific brain regions (Joris et al. 2018). This may be seen as a limitation of the technique and by the fact we included participants who had either a bilateral or unilateral signal. Since we averaged the left- and right-side measures we may be reporting a reduced CVR in certain tasks. However, we captured the change in CVR without the exclusion of unilaterality, and, therefore, may not be a limitation, because this may have underestimated the associations between primary outcomes and the covariates. Additionally, it would not be expected that training status influences changes unilaterality, rather globally. More highly localised and direct processes could be used in future studies, if available, and these may give more definitive results. However, it is unlikely that this should be a limiting factor given that correlations between direct methods of CBF determination and TCD have been positively associated and validated (Miyazawa et al. 2018; Willie et al. 2011).

What was interesting in this study and has already been highlighted as a potential limitation above, is that the trained group had a higher level of education than the untrained. While others have reported this previously (Dishman et al. 1985; Lawrence 2017), there is also conjecture if this is limited to major metropolitan areas and extends to regionals areas. In Australia, particularly Queensland, metropolitan areas tend to be both more educated and physically active (QHealth 2020). It may be that educational level is a mediator of performing exercise, as education may be an important mediator of motivation to perform exercise (Dishman et al. 1985; Lawrence 2017). Alternatively, it may be that participating in exercise promotes or motivates individuals to further their education (Dishman et al. 1985) However, this differs regionally and rurally, where those in regional areas may be more educated than some areas but are less physically active than areas of lower education (QHealth 2020). In these areas, education level may not mediate exercise participation. This may account for why there were differences in education level in our participants, as we recruited a mix of people from metropolitan and regional areas. Determining sociodemographic differences were outside the scope of this study but may be an opportunity for future studies to perform these analyses within a single location and compare the differences between metropolitan, regional and rural centres.

A greater adiposity may contribute to a reduced cerebrovascular and cognitive function, independent of exercise training status (Bangen et al. 2014; Toda 2012; Toth et al. 2017; Woods et al. 2011). Obesity and sedentary behaviour rates are growing and may lead to the development of comorbidities, including cognitive decline and dementia (Taylor 2014; Brown et al. 2017; Beydoun et al. 2008). The untrained group in our study had a higher body mass, hip circumference, waist circumference, total body fat and hip-to-waist ratio than the trained group. We did not match the groups by body mass, BMI, waist circumference or body composition which is different from previous studies that reported no differences in BMI between the trained and untrained groups (Ainslie et al. 2008; Barnes et al. 2013; Braz et al. 2017; Miller et al. 2018; Zhu et al. 2013). We chose to not undertake this because our aim was to compare differences between older adults that had undertaken regular aerobic exercise training and those that were sedentary and untrained. As a result, the sedentary and untrained individuals had a higher a central adiposity and this was reflective of sedentary individuals in the regional areas of Australia where they were recruited. Given the high rates of physical inactivity and obesity globally, our aim was to replicate the real-world scenario and replicate this by comparing the two groups used in this study (OECD 2020). Additionally, there are variations in hypercapnia protocols, including breath-holding and manual manipulation, step-wised induction against non-stepwise induction and variation in baseline breathing (Ainslie et al. 2008; Barnes et al. 2012, 2013; Braz et al. 2017; Brown et al. 2010; Marley et al. 2020; Miller et al. 2018; Rogers et al. 1990, 1985; Zhu et al. 2013). We chose to use 5% carbon dioxide to induce hypercapnia, as this is a reliable challenge that we have undertaken previously (Evans et al. 2017a; Wong et al. 2016a, 2016b, 2016c). Finally, we acknowledge that our data may be confounded by other sources of variation such as diet and sex differences between participants. However, we attempted to minimise the latter by matching each group with an equal number of men and women.

## Conclusion

In conclusion, older adults that had undertaken regular aerobic exercise training over the past 40 years had higher cerebrovascular and cognitive functions than age and sex matched, untrained and sedentary adults and their overall cognitive performance correlated moderately with their CVR to hypercapnia and strongly with their CVR to cognitive stimuli. However, following adjustment for covariates, CVR to hypercapnia, CVR to cognitive stimuli and total composite cognitive score did not differ significantly between trained and untrained groups. Our novel data demonstrate that there is a relationship between cerebrovascular and cognitive function in older adults and that there is an interaction between exercise training and cardiometabolic factors that may directly influence cerebrovascular and cognitive functions. Future studies are needed to examine the specific role of each these variables and how they are modulated by exercise to maintain cerebrovascular and cognitive function in older adults.

## Supplementary Information

Below is the link to the electronic supplementary material.

Supplementary file1 (DOCX 15 KB)

## Data Availability

The datasets generated during and/or analysed during the current study are available from the corresponding author on reasonable request.
