# Supplementary material for: The benefits of regular aerobic exercise training on cerebrovascular function and cognition in older adults
Source: Eur J Appl Physiol. 2023 Feb 19;123(6):1323–42. doi: 10.1007/s00421-023-05154-y (PMC9938957; doi:10.1007/s00421-023-05154-y)
Supplement: Supplementary file 1 — Supplementary file1 (DOCX 15 KB) [file 421_2023_5154_MOESM1_ESM.docx]

### **Supplemental Table 1.** Types of exercise participated in and the duration of participation in years. Values are means ± SEM.

| Exercise type | Number who have participated in exercise type | Participants who have participated in exercise type (%) | Time participated in exercise type (years) |
| --- | --- | --- | --- |
| Walking activities including hiking | 12 | 92 | 32 ± 4 |
| Stair-climbing machine | 3 | 23 | 3 ± 2 |
| Jogging/running | 8 | 62 | 21 ± 5 |
| Cycling | 10 | 77 | 16 ± 4 |
| Horseback riding | 3 | 23 | 7 ± 2 |
| Dancing, gymnastics and rhythmic exercises | 5 | 38 | 32 ± 5 |
| Swimming | 10 | 77 | 12 ± 4 |
| Rowing/paddling based exercises | 8 | 62 | 11 ± 4 |
| Skiing (downhill, cross-country, water, machine) | 1 | 8 | 5 ± 0 |
| Tennis and other racquet sports | 9 | 69 | 18 ± 4 |
| Ball sports (e.g. basketball, netball, bowling) | 2 | 15 | 8 ± 2 |
| Surfing | 1 | 8 | 34 ± 0 |
| Golf (walking, no golf cart) | 4 | 31 | 9 ± 4 |
